# The Impact of Liver Metastasis on Anti-PD-1 Monoclonal Antibody Monotherapy in Advanced Melanoma: Analysis of Five Clinical Studies

**DOI:** 10.3389/fonc.2020.546604

**Published:** 2020-09-29

**Authors:** Xuan Wang, Qing Ji, Xieqiao Yan, Bin Lian, Lu Si, Zhihong Chi, Xinan Sheng, Yan Kong, Lili Mao, Xue Bai, Bixia Tang, Siming Li, Li Zhou, Chuanliang Cui, Jun Guo

**Affiliations:** Key Laboratory of Carcinogenesis and Translational Research (Ministry of Education), Department of Renal Cancer and Melanoma, Peking University Cancer Hospital and Research Institute, Beijing, China

**Keywords:** melanoma, liver metastasis, prognosis, immunotherapy, programmed cell death protein 1

## Abstract

Anti-programmed cell death protein 1 (PD-1) monoclonal antibody therapy is becoming a standard treatment for advanced melanoma that produces durable responses and prolonged survival, but the prognosis of patients with liver metastases is still unsatisfactory. Here, we analyzed five clinical studies (second-line or later, JS001-I-PK, CT4, KN151, BGB-A317-102, and SHR-1210-102; performed between 2015 and 2018) of anti-PD-1 monotherapy for advanced melanoma to explore prognostic variables for patients with liver metastases. A total of 168 patients with stage IV melanoma were included, among which 47 had liver metastasis and 121 did not. The objective response rate (ORR) of the no liver metastasis group was significantly higher than that of the liver metastasis group (20.7 vs. 4.3%, *P* < 0.05). The median progression-free survival (PFS) time was 3.6 months for the patients with liver metastasis and 7.4 months for those without liver metastasis (*P* < 0.05). The no liver metastasis group also had a longer median overall survival (OS) time than the liver metastasis group (22.8 vs. 15.7 months, *P* < 0.05). Multivariate analysis showed that liver metastasis was negatively associated with PFS. In the liver metastasis group, compared to metastases in other sites (lymph node, subcutaneous, and lung), liver metastases responded worse to anti-PD-1 monotherapy and were most likely to progress. Intrahepatic progression (defined as an increase in liver metastasis by more than 20% from baseline or having new liver metastases, *P* < 0.05) was negatively associated with OS, which indicates the need to find a more effective therapy that can target liver metastases. Interestingly, with a median PFS and OS time of 6.0 and 30.9 months, respectively, previous oncolytic virotherapy might bring more benefits to patients with liver metastasis, but confirmation is needed because of the limited number of samples. These findings emphasize that liver metastasis is a poor prognostic factor for advanced melanoma treated with anti-PD-1 monotherapy. Further exploration is still needed to find a new treatment approach for these patients.

## Introduction

Programmed cell death protein 1 (PD-1) is a highly expressed protein on tumor-infiltrating lymphocytes that reduces the activity of T cells and blocks the immune response upon interaction with programmed death ligand-1 on the surface of tumor cells, thus leading to immune escape (1). Currently, PD-1 is an important target in immunotherapy treatment for melanoma (2–4). Given their abilities to produce durable responses and prolonged survival, two anti-PD-1 monoclonal antibodies, pembrolizumab and nivolumab, have been approved in the United States for use in advanced melanoma (5–11). In phase 1b clinical trials of pembrolizumab including 655 patients with advanced melanoma, the overall objective response rate (ORR) was 33%, and the median overall survival (OS) time was 23 months (7). With a median OS time of 16.8 months, nivolumab produced an ORR of 31% in a study of 107 patients with advanced melanoma (9).

However, because anti-PD-1 monotherapy is associated with reduced response and shortened progression-free survival (PFS), its clinical effects on melanoma patients with liver metastases are unsatisfactory (12). Here, we summarize five clinical studies for advanced melanoma performed at our clinical research center between 2015 and 2018, describing the clinical characteristics of patients with melanoma liver metastases treated with anti-PD-1 monoclonal antibody therapy, trying to explore possible prognostic factors.

## Materials and Methods

### Study Design

From 2015 to 2018, 187 patients at Peking University Cancer Hospital were enrolled in five clinical studies of anti-PD-1 monoclonal antibody monotherapy for advanced melanoma (second-line or later, JS001-I-PK, CT4, KN151, BGB-A317-102, and SHR-1210-102), and all the patients with stage IV disease (AJCC Cancer Staging Manual 8th ed.) were included in this retrospective cohort study, for a total of 168 included patients.

### Statistical Analysis

Descriptive analysis was used to describe the demographic and clinical characteristics of the liver metastasis and no liver metastasis groups, and consistency was compared by the Kruskal–Wallis test for continuous variables and the Chi-square test for categorical variables. Univariate and multivariate Cox regression analyses were used to identify factors associated with PFS and OS, estimating their hazard ratios and associated 95% confidence intervals. Median OS and PFS and their associated 95% confidence intervals were calculated by the Kaplan–Meier method. Statistical analyses were performed using SPSS V22 (IBM Corp., Armonk, NY) and GraphPad PRISM (Prism 8.0.2; GraphPad Software, LLC). All tests were two-sided, with *P*-values < 0.05 considered statistically significant.

## Results

### Baseline Clinical Characteristics

A total of 168 patients with stage IV metastatic melanoma who received anti-PD-1 monoclonal antibody monotherapy (second-line or later) were studied. The age of the patients ranged from 22 to 77 years old, and most of the patients were younger than 65 years old. There were slightly fewer male patients than female patients. Acral melanoma was the most common type (38.1%), and the most common metastatic site was the lung (44.6%). Nearly half of the patients had an Eastern Cooperative Oncology Group (ECOG) performance status of 0, and approximately one-third of them had a baseline LDH level higher than the normal level. Twenty-seven patients had BRAF V600 mutations (Table 1).

**TABLE 1:** Baseline clinical characteristics and consistency of patients with and without liver metastases.

Characteristics	*n* (%)	Liver + (%) (*n* = 47)	Liver − (%) (*n* = 141)	*P*-value
**Age**				
<65	143 (85.1)	41 (87.2)	102 (84.3)	*P* = 0.489*
≥65	25 (14.9)	6 (12.8)	19 (15.7)	
**Sex**				
Male	74 (44)	22 (46.8)	52 (43)	*P* = 0.653
Female	94 (56)	25 (53.2)	69 (57)	
**ECOG performance status**				
0	76 (45.2)	19 (40.4)	57 (47.1)	*P* = 0.08
≥1	92 (54.8)	28 (59.6)	64 (52.9)	
**LDH level**				
Normal	110 (66.5)	24 (51.1)	86 (71.1)	*P* = 0.014
Elevated	58 (34.5)	23 (48.9)	35 (28.9)	
**Primary site of melanoma**				
Acral	64 (38.1)	15 (31.9)	49 (40.5)	*P* = 0.364
Cutaneous	46 (27.4)	11 (23.4)	35 (28.9)	
Mucosal	31 (18.5)	12 (25.5)	19 (15.7)	
Unknown	27 (16.1)	9 (19.1)	18 (14.9)	
**Lung metastasis**				
No	75 (44.6)	19 (40.4)	56 (46.3)	*P* = 0.493
Yes	93 (55.4)	28 (59.6)	65 (53.7)	
**Brain metastasis**				
No	165 (98.2)	46 (97.9)	119 (98.3)	*P* = 0.835
Yes	3 (1.8)	1 (2.1)	2 (1.7)	
**BRAF V600**				
Wild-type	128 (76.2)	39 (83)	89 (73.6)	*P* = 0.099
Mutated	27 (16.1)	4 (8.5)	23 (19.0)	
Unknown	13 (7.7)	4 (8.5)	9 (7.4)	
**Objective response**				
Yes	27 (16.1)	2 (4.3)	25 (20.7)	*P* = 0.009
No	141 (83.9)	45 (95.7)	96 (79.3)	

*The consistency of age was compared by the Kruskal–Wallis test. That of other variables was compared by the Chi-square test.

Dividing the patients into two groups based on the presence or absence of liver metastases resulted in 47 patients in the liver metastasis group and 121 patients in the no liver metastasis group. There were no significant differences between the two groups in terms of sex, age, ECOG performance status, primary tumor location, BRAF mutation, or extrahepatic metastases, but the levels of LDH were unevenly distributed (Table 1). The liver metastasis group included a higher proportion of patients with an elevated baseline LDH level.

### Overall Analysis

As of November 2019, 98 of the 168 patients, including 32 who had liver metastases, had died, accounting for 68.1% of the patients in the liver metastasis group. Among the 141 patients with progressive disease (PD), 39 had liver metastases, accounting for 83% of the liver metastasis group. There were 27 patients who achieved an objective response (OR) as their best response, and only 2 of these patients had liver metastases. Thus, the ORR of all patients was 16.1%. In the liver metastasis group, the ORR was 4.3%, which was significantly lower than that in the no liver metastasis group (20.7%, *P* < 0.01).

**FIGURE 1:**
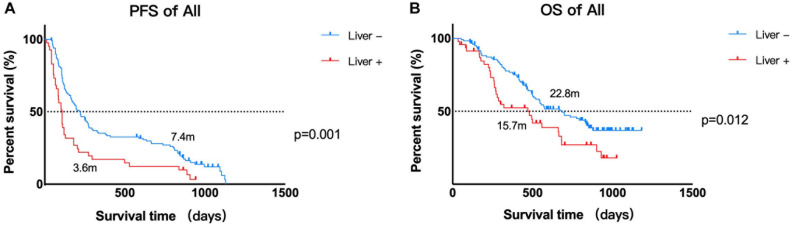
Kaplan–Meier estimates of the PFS and OS of patients. **(A)** Kaplan–Meier estimate of the PFS of 168 patients. Median PFS time: liver metastasis vs. no liver metastasis, 3.6 vs. 7.4 months, *P* = 0.002. **(B)** Kaplan–Meier estimates of the OS of 168 patients. Median OS time: liver metastasis vs. no liver metastasis, 15.7 vs. 22.8 months, *P* = 0.016.

**FIGURE 2:**
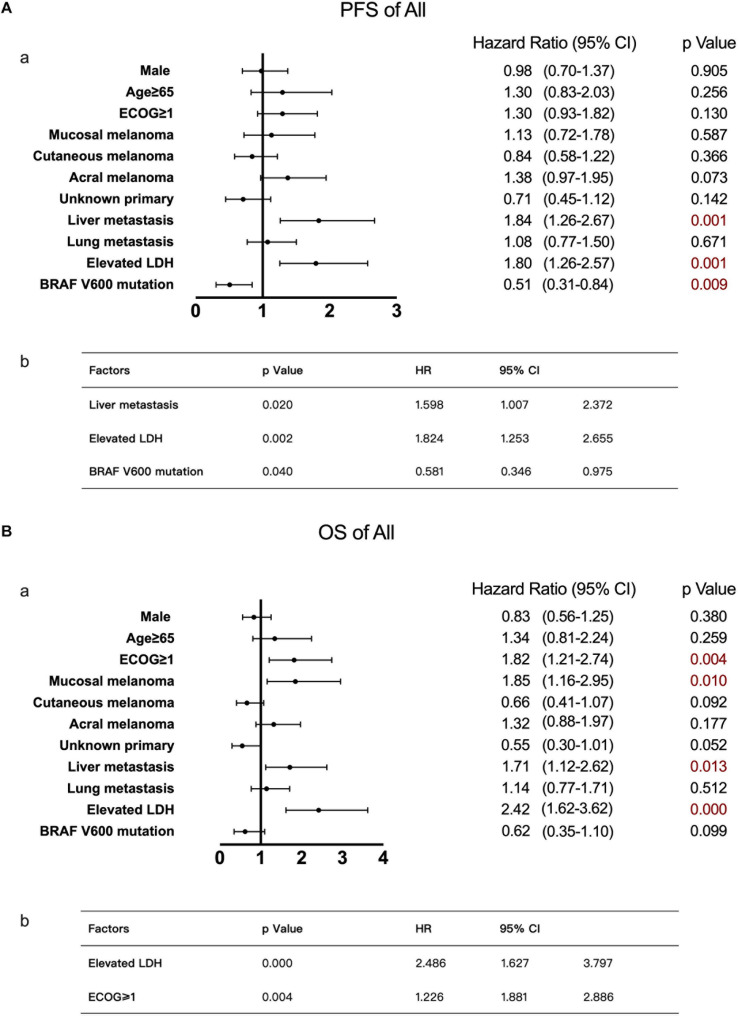
Univariate and multivariate analyses of 168 patients with advanced melanoma. The results of univariate analyses are shown in forest plots; hazard ratios and associated 95% confidence intervals were calculated for each subgroup and are illustrated by the dotted vertical line. Statistical significance is depicted in the right column. The results of multivariate analyses are shown in tables; the *P*-value, hazard ratios and associated 95% confidence intervals are listed. **(A)** Univariate (a) and multivariate (b) analyses of PFS. **(B)** Univariate (a) and multivariate (b) analyses of OS.

As calculated by the Kaplan–Meier method, the median PFS time of the 121 patients without liver metastases was 7.4 months, while that of patients with liver metastases was 3.6 months (*P* < 0.05, Figure 1A). Univariate analysis revealed that the factors associated with PFS in advanced melanoma were risk factors, including liver metastases and baseline LDH levels, and a protective factor, BRAF V600 mutations. Based on these data, we constructed a multivariate model using hazard Cox regression, which produced the same conclusion. According to the hazard ratios, liver metastasis and elevated baseline LDH levels had similar effects on patients’ PFS, with an elevated LDH level being more closely associated with shorter PFS than was liver metastasis (Figure 2A).

Using the same method described above, the median OS time of patients in the no liver metastasis group was 22.8 months, which was significantly longer than that of the patients in the liver metastasis group (15.7 months, *P* < 0.05, Figure 1B). Univariate analysis showed that, in addition to the liver metastasis status, the baseline LDH level, disease subtype, and ECOG performance status were also correlated with OS. By multivariate analysis after the adjustment for covariates, liver metastasis was no longer associated with OS. The other covariates that were significantly correlated with OS were the ECOG performance status and baseline LDH level (Figure 2B).

### Analysis of Patients With Liver Metastases

**FIGURE 3:**
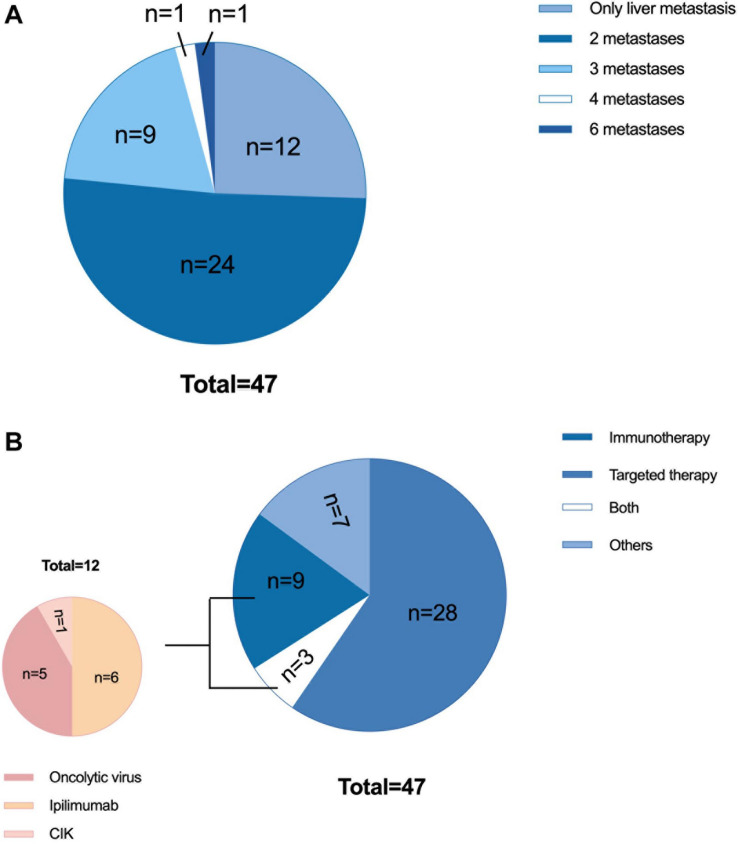
Characteristics of 47 patients with liver metastases. **(A)** Previous therapy: 31 patients received targeted therapy (including chemotherapy or chemotherapy combined with antiangiogenic therapy), 12 patients received immunotherapy, 3 patients received both targeted therapy and immunotherapy, and 7 patients did not receive either immunotherapy or targeted therapy. Of the 12 patients who received immunotherapy, 6 were treated with ipilimumab, 5 were treated with oncolytic virotherapy, and 1 was treated with autologous CIK immunotherapy. **(B)** Tumor burden: 12 patients had only liver metastases, 24 patients had metastases in two organs, 9 patients had metastases in three organs, 1 patient had metastases in four organs, and 1 patient had metastases in six organs. CIK, cytokine-induced killer cells.

Of the 47 patients with liver metastases, the sex distribution was roughly balanced, and the majority of patients were younger than 65 years old. Twenty-three patients had an elevated baseline LDH level, accounting for approximately half of the group. There were 34 patients had other organ involvements aside from liver and a total of 51.1% of patients had metastases in two organs (Figure 3A). A total of 63.8% of the patients received anti-PD-1 monoclonal antibody therapy as second-line therapy, and 36.1% received it as third-line or later therapy. In terms of prior systemic antitumor therapy, thirty-one patients had received targeted therapy, and 12 had received immunotherapy [including six treated with ipilimumab, five treated with oncolytic virotherapy, and one treated with cytokine-induced killer cell (CIK) immunotherapy, Figure 3B].

**FIGURE 4:**
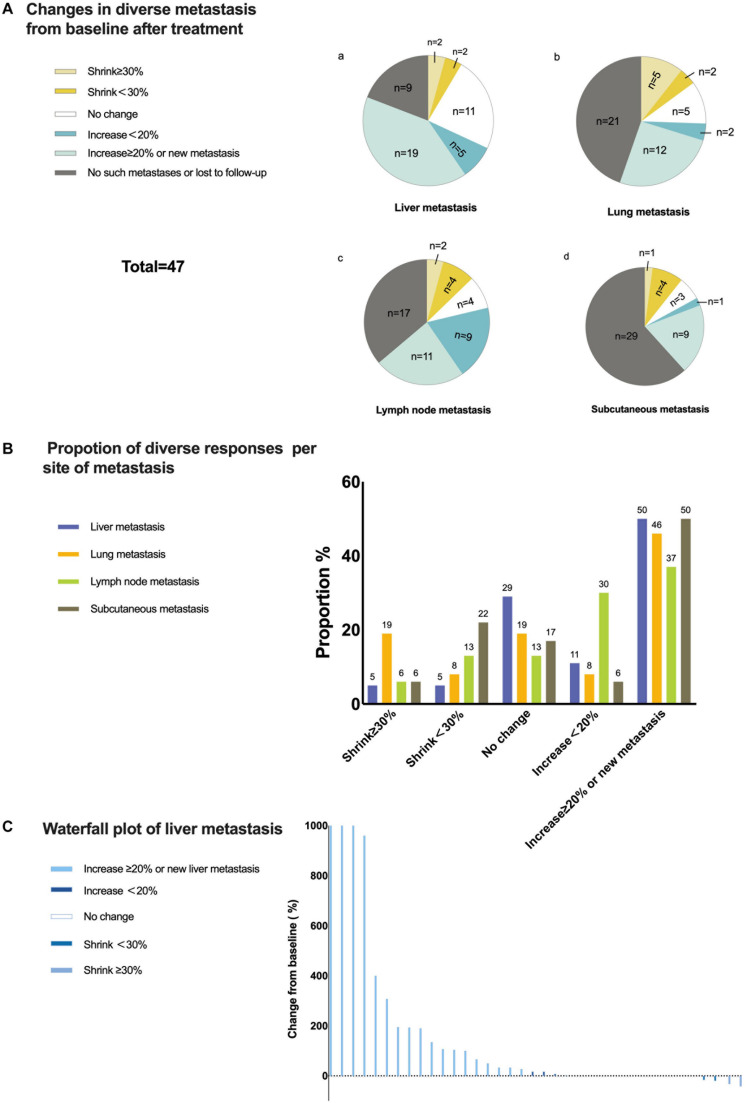
Responses of liver metastases to treatment. **(A)** Changes in diverse metastases from baseline to after treatment. Only changes in the same lymph node drainage area were taken into consideration for lymph node metastasis and subcutaneous metastasis. **(B)** Proportion of diverse responses per metastatic site. Each number above the bar is the value of the proportion. **(C)** Waterfall plot of the percentage change in liver metastasis sizes from baseline after treatment for each patient.

As mentioned before, with only two patients achieving an OR in the liver metastasis group, the ORR was only 4.3%. To determine the responses of different metastases to anti-PD-1 monoclonal antibody monotherapy, we analyzed changes in metastases within and outside the liver relative to the baseline. Liver metastases had a poor response to treatment. Until the cut-off time, only four cases of liver metastasis shrinking occurred. However, nearly half of the liver metastases increased, and most of them increased by more than 20% or generated a new liver metastasis. Based on the immune-related Response Evaluation Criteria in Solid Tumors, for the same organ or lymph node drainage area, shrinkage ≥30% was considered a significant reduction, and an increase ≥20% or a new metastasis was considered a significant increase. Except for lymph node metastases, almost half of the cases of other kinds of metastases, including lung metastasis and subcutaneous metastasis (other organ metastases were not included because of the low sample numbers), increased significantly if invalid data were removed. Approximately 27% of lung metastases shrunk after treatment, and 19% shrunk significantly. This indicated that lung metastases might be more likely to be suppressed by anti-PD-1 monoclonal antibody monotherapy. However, from the perspective of tumor shrinkage or growth, liver metastases consistently showed the least benefits. This is illustrated more clearly in the waterfall plot showing the changing size of liver metastases (Figure 4). In addition, among 21 patients with extrahepatic metastases in which liver metastases increased, six cases (28.6%) showed only liver metastasis growth, while five had extrahepatic metastasis shrinkage, and another had no change from baseline.

**FIGURE 5:**
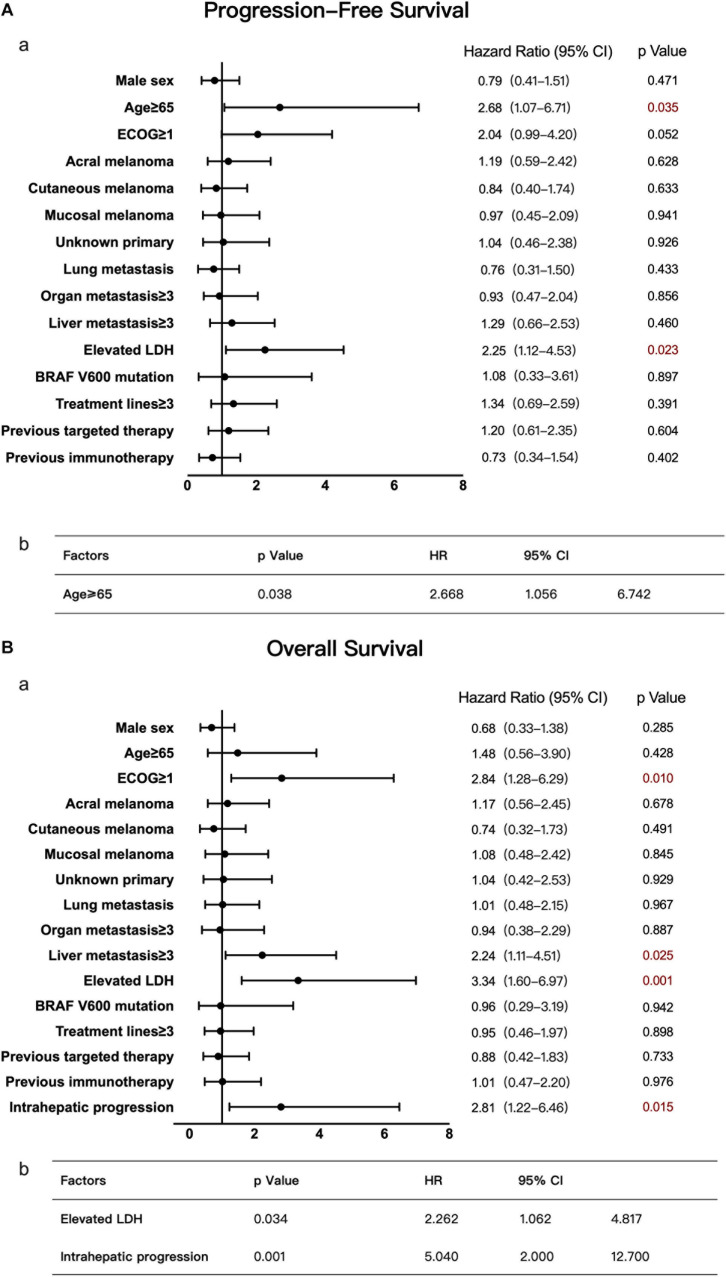
Univariate and multivariate analyses of 47 patients with liver metastases. The results of univariate analyses are shown in forest plots; hazard ratios and associated 95% confidence intervals were calculated for each subgroup and are illustrated by the dotted vertical line. Statistical significance is depicted in the right column. The results of multivariate analyses are shown in tables; the *P*-value, hazard ratios and associated 95% confidence intervals are listed. **(A)** Univariate (a) and multivariate (b) analyses of PFS. **(B)** Univariate (a) and multivariate (b) analyses of OS.

Univariate analysis of this group suggested that the baseline LDH level and age were risk factors for PFS. Multivariate analysis confirmed that an age ≥65 was significantly and negatively associated with the PFS of patients with liver metastases (Figure 5A), with a much shorter median PFS time seen in older patients than in younger patients (age ≥ 65 vs. age < 65, 1.9 vs. 3.7 months, *P* < 0.05). In terms of OS, univariate analysis suggested that the ECOG performance status, LDH level, number of liver metastases, and condition of liver metastasis progression affected OS. By multivariate analysis, an elevated baseline LDH level and intrahepatic progression (defined as an increase in liver metastasis by more than 20% from baseline or having new liver metastases) were demonstrated to be independent risk factors (Figure 5B). The median OS time of patients with elevated LDH levels was only 9.3 months, while that of patients with normal LDH levels was 22.7 months. Patients with intrahepatic progression had a median OS time of 10.6 months, while patients without intrahepatic progression had a median OS of 30 months (*P* < 0.05).

There were no apparent differences between patients who received previous immunotherapy and those who did not, according to the univariate analyses of PFS and OS. However, large gaps in median PFS and OS appeared among patients receiving different immunotherapies. The six patients receiving previous ipilimumab had a median PFS time of 2.8 months and a median OS time of 6.5 months, while the five patients who had received oncolytic virotherapy (twp treated by cutaneous injection and three treated by intrahepatic injection) had an obviously longer median PFS time of 6 months and a longer median OS time of 30.9 months. For the two patients receiving cutaneous injection, their PFS times were 1.8 and 3.6 months, and their OS times were 9.3 and 23.4 months. For those receiving intrahepatic injection, their PFS times were 6.0, 9.1, and 28.8 months, and their OS times were 15.7, 28.8, and 30.9 months, respectively. These findings implied that anti-PD-1 monoclonal antibody therapy may produce different outcomes when combined with diverse immunotherapies. Unfortunately, the type of prior immunotherapy was not included in the univariate and multivariate analyses because of the small sample size.

## Discussion

In this article, we investigated the clinical characteristics of 168 patients receiving anti-PD-1 monoclonal antibody monotherapy to explore the effects of liver metastasis on patient prognosis. It was discovered that the prognosis of patients with liver metastases was relatively poor, with median PFS and OS times significantly shorter than those of patients in the no liver metastasis group. In the liver metastasis groups, there were only two patients achieving an OR, with an ORR of 4.3%, which was much lower than the 20.7% ORR found in the no liver metastasis group. Different from most studies of melanoma, acral, and mucosal melanoma with known poorer prognosis account for most subtypes in this study, which may affect the generalizability of the results to a population with predominantly superficial spreading and nodular melanomas. However, such a distribution in our study is more typical in Asian, and the results could be very meaningful for Asian patients.

Supporting the findings described above, multivariate analysis also indicated that liver metastasis was a risk factor for disease progression. In addition, specific analysis of different metastases revealed that liver metastasis responded worse to anti-PD-1 monotherapy than other metastases. Subsequent analysis showed that the OS of patients with intrahepatic progression was much shorter, only 10.6 months, than that of patients without intrahepatic progression, suggesting the importance of inhibiting the growth of liver metastases. In a clinical study of melanoma patients treated with ipilimumab combined with anti-PD-1 therapy, liver metastases also had the lowest lesional response rate and an inferior ORR, PFS, and OS (13), which was similar to our conclusion.

To explore possible prognostic factors, statistical analysis of the liver metastasis group was performed. The results suggested that age was associated with PFS, and an elevated baseline LDH and intrahepatic progression were negatively associated with OS. The median OS of the patients with normal LDH levels in the liver metastasis group was 22.7 months, which was almost equal to that of patients in the no liver metastasis group. Therefore, for patients with a normal LDH level, a conclusion might be drawn that they can still try anti-PD-1 monotherapy despite the presence of liver metastases.

Currently, anti-PD-1 monoclonal antibody therapy is an important treatment for advanced melanoma that has achieved relatively positive effects. However, as shown in this article, for patients with liver metastases, monotherapy dose not result in satisfying outcomes. There is still no exact explanation for the poor effects of anti-PD-1 monotherapy on liver metastasis. Some contributed this to local immune tolerance in the liver, which has been observed in orthotopic liver transplantation (12). The effects of PD-1 blockade therapy may be enhanced by modulating the immune microenvironment of the liver. In colorectal cancer mice with liver metastasis, blockade of TGF-β signaling, which is an important factor in the microenvironment in liver metastasis, has been found to rendered tumors susceptible to anti-PD-1 therapy (14–17).

As mentioned above, the PFS and OS of the five patients (two treated by cutaneous injection and three treated by intrahepatic injection) who received oncolytic virotherapy were obviously prolonged, especially for the three patients who received intrahepatic oncolytic virotherapy. Oncolytic viruses have been demonstrated to recruit tumor-infiltrating lymphocytes to the tumor microenvironment, and as a result, they can alter immune-desert and immune-excluded tumor landscapes (18). Increased CD8^+^ and CD4^+^ T cell numbers, elevated PD-L1 protein expression and IFN-γ gene expression have been observed in patients receiving a combination of oncolytic virotherapy and immune checkpoint blockade therapy (19, 20). At present, the oncolytic virotherapy talimogene laherparepvec combined with pembrolizumab (phase 1b clinical trial, ORR 62%) or ipilimumab (phase II clinical trial, ORR 39%) has been proven to have better effects than monotherapy (19, 21). A large randomized phase III trial is also underway to evaluate oncolytic virotherapy combined with pembrolizumab in comparison to pembrolizumab alone (KEYNOTE-034). We speculated that the favorable outcomes of the five patients, especially the prolonged survival of the three who received intrahepatic injection, which still need prospective studies to validate, might be a result of modulation of the local immune microenvironment in the liver. Intrahepatic injection of an oncolytic virus is likely to be a promising way to improve the effects of anti PD-1 immunotherapy and deserves further research.

There are some limitations to this study that must be acknowledged. First, as mentioned above, there is a lack of generalizability to the population with predominantly superficial spreading and nodular melanomas because of the over-representation of acral and mucosal melanoma. Besides, all patients were rigorously screened according to the inclusion and exclusion criteria of clinical trials rather than enrolled randomly, also leading to a lack of generalizability to some extent. For example, the percentage of patients with brain metastasis was unexpectedly low, which would affect the generalizability of the results to those without liver metastasis. Additionally, with a total sample size of 168 patients and only 47 patients with liver metastasis, the number of patients was insufficient. Additional studies with a larger sample size are needed to validate and complement our conclusions.

From the analysis above, liver metastasis might be a poor prognostic factor for advanced melanoma treated with anti-PD-1 monotherapy. Further exploration is still needed to investigate the potential mechanism and find new treatment approaches for these patients.

## Data Availability Statement

The raw data supporting the conclusions of this article will be made available by the authors, without undue reservation.

## Ethics Statement

The studies involving human participants were reviewed and approved by the Beijing Cancer Hospital Institutional Review Board. All participants provided written informed consent.

## Author Contributions

JG and CC: research concept and design. XW and QJ: data collection, data analysis and interpretation, and writing the manuscript. All authors read and approved the final manuscript.

## Conflict of Interest

The authors declare that the research was conducted in the absence of any commercial or financial relationships that could be construed as a potential conflict of interest.
